# Role of Ca^2+^ doping on the enhancement of dielectric properties of Sr_2–x_Ca_x_NiWO_6_ for energy storage device application

**DOI:** 10.1038/s41598-023-28296-7

**Published:** 2023-01-23

**Authors:** Nur Amira Farhana Mohamed Saadon, Nurul Izza Taib, Chee Wah Loy, Zakiah Mohamed

**Affiliations:** 1 Faculty of Applied Sciences, Universiti Teknologi MARA, 40450 Shah Alam, Selangor Malaysia; 2 Faculty of Applied Sciences, Universiti Teknologi MARA, Perak Branch, Tapah Campus, 35400 Tapah Road, Perak Malaysia; 3 ARC Training Centre for Automated Manufacture of Advanced Composites, University of New South Wales, Sydney, NSW 2052 Australia

**Keywords:** Materials science, Physics

## Abstract

In this paper, Sr_2–x_Ca_x_NiWO_6_ (x = 0.00, 0.02, 0.04, 0.06) were synthesized using a solid-state reaction method. The crystal structure, optical and dielectric properties of the compounds were examined using X-ray diffraction (XRD), scanning electron microscope (SEM) with energy dispersive (EDX) analysis, Ultraviolet–visible (UV–vis) diffuse reflectance spectroscopy and electrochemical impedance spectroscopy respectively. The Rietveld refinement of XRD confirmed that the compounds crystallized in a tetragonal structure with a space group I4/m. According to the SEM images, the grain sizes of the compounds decreased as the dopant increased. The UV–vis analysis revealed that the band gap energy of the compounds decreased from 3.17 eV to 3.13 eV as the amount of doping increased from x = 0.00 to x = 0.06. A dielectric characterization showed that the dielectric constant (ε′) and dielectric loss (tan δ) for all compounds possessed a similar trend where it was higher in low-frequency area (~ 1 Hz) and dropped instantaneously with the enhancement of frequency up to 1 MHz until it reached constant values.

## Introduction

Studies on double perovskite compounds have attracted huge attention of numerous researchers due to its diversity of structural, magnetic and electrical properties^1–3^. Due to the existence of these characteristics, double perovskites are widely sought-after in the development of a variety of applications, such as low field magneto resistive sensors, magnetic random access memories (MRAM)^4^, microwave devices^5^ and solar cells^6^. Generally, double perovskites which are derived from conventional perovskites, ABO_3_^7^ have a general formula AA’BB’O_6_ or A_2_BB’O_6_ where A is categorized as alkaline earth metals such as Sr, Ca and Ba, B = Fe, Co, Ni, Cr and B’ = Mo, W, Re, U are transition metal ions. In these compounds, A site cations are the largest atom compared to B site cations which are 12-fold coordinated meanwhile B and B’ cations are six-fold coordinated to the oxygen. Depending on the components of the A and B sites, these compounds’ crystallographic structure, magnetic and electrical characteristics can differ significantly. A small chemical substitution in these compounds can lead to a distortion of crystal structure which are connected to their electrical behavior^8^. Additionally, the structural and magnetic properties of the compounds also can be tuned by adjusting the size of the cations or the number of d electrons of the B site cations^9^.

Recently, tungstate-based double perovskite materials, A_2_BWO_6_ (A = Ba, Sr, Ca and B = Mg, Ni, Co, Zn) have caught the attention of researchers and have been the subject of considerable study for their complete 1:1 ordered state due to greater disparities in ionic radii and charge of B site cations. Previous study on the structural properties of strontium tungstate-based double perovskite, Sr_2_NiWO_6_ reported that the compound crystallized in a tetragonal structure with a space group I4/m. However, gradually increasing the size of the A site cation by substituting the alkaline earth cation of Sr^2+^ with Ba^2+^ resulting in a structural phase transition from tetragonal to cubic^10^. In addition, Alsabah et al., also claimed that their research showed a structural phase shift from cubic (Fm$\overline{3}$m) to monoclinic (P21/n) phase when Sr^2+^ was doped at the A site in Ba_2–x_Sr_x_ZnWO_6_ (1.00 ≤ x ≤ 2.00)^6^. Apart from that, it was reported in a different study that Ca_2_NiWO_6_ had a monoclinic structure with the space group P21/n^11^.

The studies on how different A and B site ions affect the optical characteristics of double perovskites have also received a lot of attention. For example, increasing substitution of Ba^2+^ by Sr^2+^ in Ba_2–x_Sr_x_ZnWO_6_ showed that the band gap energy increased from 3.52 to 3.7 eV. The reason of this occurrence may be attributed to the decreasing of ionic radius which caused the band gap energy to increase. In a different study on Mg_2_YVO_6_ and Sr_2_YVO_6_ compounds, it was discovered that the band gap energy decreased from 2.9 to 2.48 eV as the size of the ionic radius of the A site atoms increased. This finding highlight that the increase in ionic radius reduced the excitation distance for the electrons from valence to conduction band^12^. In addition, the band gap energy obtained for Ba_2_MgWO_6_ and Ba_2_ZnWO_6_ were 3.4 eV and 3.5 eV respectively^13^. From these results, the compounds were classified as wide band gap semiconductor materials.

The dielectric properties of double perovskites also were investigated which this characteristics can be related to a number of factors, including the tolerance factor, size, density and polarizability impact. According to a recent work, the dielectric measurement at frequency of 0.1 kHz were performed on Ba_2_NiWO_6_ double perovskite which possessed high values of dielectric constant, 290 compared to Ba_2_NiTeO_6_ which at 96^14^. The value of dielectric constant was higher as W^6+^ has an effective charge which improves the conduction of electrons in the compound. Other than that, Bijelic et al.^15^, reported that the dielectric constant values of several B site doped materials, Sr_2_NiWO_6_ and Sr_2_NiTeO_6_ (341 and 308, respectively) and dielectric loss (0.06 and 0.23, respectively). Since the dielectric loss of tungsten, W^6+^ compound was substantially lower than Te^6+^ based compound, thus the compound is promising for electronic devices applications.

Despite the fact that a number of works have been reported on tungstate based double perovskites, to the best of our knowledge, there is still no research or study done on the structural, optical and dielectric properties of Ca^2+^ doping at the Sr^2+^ site of Sr_2_NiWO_6_ double perovskite. The Sr_2–x_Ca_x_NiWO_6_ (x = 0.00, 0.02, 0.04, 0.06) double perovskite was synthesized using solid-state reaction method and the effect of Ca^2+^ dopant on the structural, optical and dielectric properties of the compounds were studied using X-ray diffraction (XRD), scanning electron microscope (SEM) with energy dispersive (EDX) analysis, Ultraviolet–visible (UV–vis) spectroscopy and electrochemical impedance spectroscopy (EIS) respectively.

## Experimental

A solid state reaction technique was used to create the compound Sr_2–x_Ca_x_NiWO_6_ (x = 0.00, 0.02, 0.04, 0.06). Strontium carbonate (Sr_2_CO_3_), calcium carbonate (CaCO_3_), nickel oxide (NiO) and tungsten oxide (WO_3_) were all obtained from Alfa Aesar and employed as initial materials due to their high purity (> 99.9%). Using an agate mortar and pestle, stoichiometric amounts of the components were combined and pounded into uniform particles over the course of two hours. The combined powders were then placed on alumina crucibles and heated in a furnace at 900 °C for 24 h at a rate of 15 °C per minute to dissolve the carbonates. The combined powders were then reground into fine powders and recalcined for 24 h at 1100 °C to improve the purity of the samples and remove all secondary phase^16,17^. The combined powders were shaped into pellets with diameters of 13 mm and thicknesses of 2.5 mm using a hydraulic press with a pressure of 5 tones. The pellets were sintered at 1100 °C for 24 h and then underwent a few characterizations. The structures of the samples were studied in an X-ray diffractometer (PANalytical model X’pert PRO MD) with copper K-alpha (Cu-Kα) radiation with λ = 1.5406 Å. The data were gathered from the samples at scattering angles ranging from 15° and 80° with a step size interval of 0.017° and counting time of 18 s for each step. The general structure analysis system (GSAS) programme and graphical user interface (EXP-GUI) were used to get the Rietveld refinements and structural analysis of the samples, which were then graphically represented by the visualization for electronic and structure analysis (VESTA) tool. Using LEO model 982 Gemini equipment, a scanning electron microscope (SEM) with energy dispersive X-ray (EDX) spectroscopy was used to examine the surface morphology and the composition of the raw materials. Using the UV–Vis spectrophotometer Lambda 750, Perkin Elmer, Waltham, USA equipment with a wavelength range of 200 nm to 800 nm, the UV–Vis diffuse reflectance spectra were observed at room temperature^18^. The Kubelka–Munk and the Tauc plot were used to convert the spectra to absorbance in order to calculate the compounds’ energy band gap. Last but not least, a 1286 electrochemical interface (SOLARTON) with a frequency response analyzer at frequencies ranging from 1 Hz to 1 MHz were used to identify the dielectric and impedance characteristics of the samples^19^.

## Results and discussions

### XRD analysis

**Figure 1:**
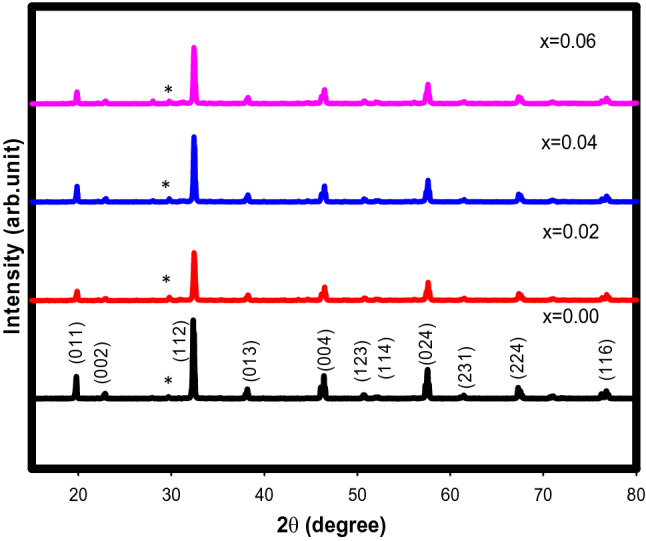
XRD patterns of Sr_2–x_Ca_x_NiWO_6_ (x = 0.00, 0.02, 0.04, 0.06) double perovskite series. An asterisk (*) sign indicates the Sr_2_WO_5_ impurity existed in the compounds.

**Figure 2:**
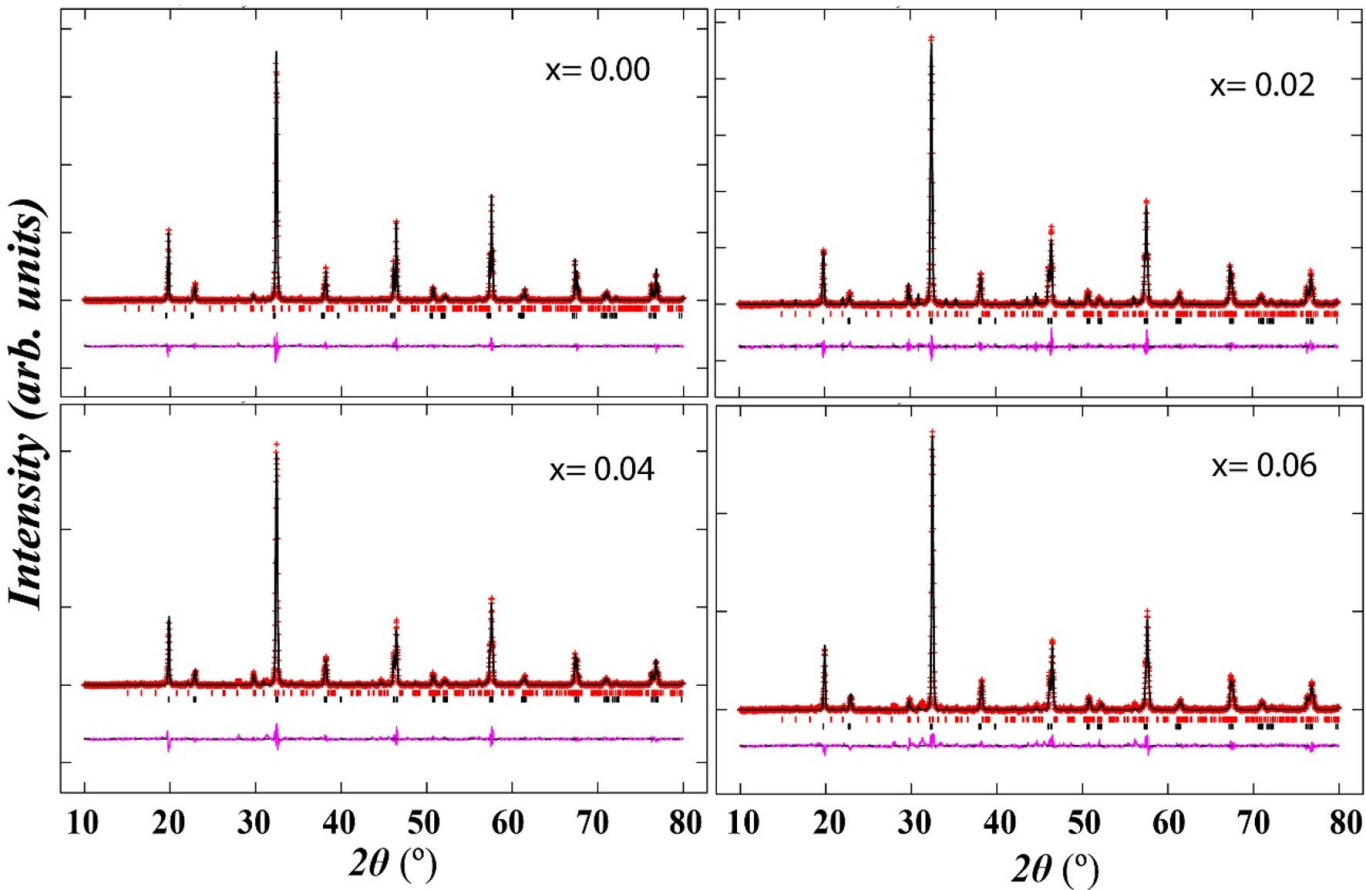
Rietveld refinement of X-ray diffraction patterns Sr_2–x_Ca_x_NiWO_6_ (x = 0.00, 0.02, 0.04, 0.06) double perovskite series. The black lines, red lines and pink lines indicated the calculated, observed and difference data respectively. Black and red ticks depicted the Bragg reflection for primary and secondary phases respectively.

Figures 1 and 2 shows the XRD patterns of Sr_2–x_Ca_x_NiWO_6_ (x = 0.00, 0.02, 0.04, 0.06) compounds and the refined data using the Rietveld refinement approach respectively. A sharp and well defined of XRD patterns were observed in Fig. 1 indicates a good crystalline perovskite phase of the samples. The diffraction peaks that were indexed to hkl plane (011), (002), (112), (013), (004), (123), (114), (024), (231), (224) and (116) matched as reported in previous studies^19,20^. All compounds formed in double phase with a small amount of Sr_2_WO_5_ impurity that present approximately at 28° and marked with asterisk (*) sign. A good agreement between the observed data and calculated line as seen in Fig. 2 indicated an acceptable refinement quality with reliabilities (χ^2^) between 1.931 and 3.396. The refinement data showed that all compounds formed in a tetragonal structure (*a* = *b* ≠ *c* and α = β = γ = 90°) of I4/m symmetry.

The equivalent data obtained from Rietveld refinement is shown in Tables 1 and 2. These compounds’ refined lattice parameters, *a*, *b* and *c* were between 5.5601–5.5692 Å, 5.5601–5.5692 Å and 7.9190–7.9267 Å respectively. The refined unit cell volumes, V were 244.889 Å^3^, 245.090 Å^3^, 245.517 Å^3^ and 245.863 Å^3^ for x = 0.00, x = 0.02, x = 0.04 and x = 0.06 correspondingly. Ideally, the cell volume should drop upon Ca^2+^ doping since the ionic radius of Ca^2+^ (1.34 Å) is smaller than that of Sr^2+^ (1.44 Å), however in the current work, the cell volume has grown. This result suggests that Ca^2+^ doping might have caused bonds in the A and B sites to elongate which increased the unit cell volume of the compounds. The stability of these compounds was calculated using Goldschmidt tolerance factor (τ) using the following Eq. (1);

1 $$\uptau = \frac{{ \frac{{{\text{r}}_{{\text{A}}} }}{2} + \frac{{{\text{r}}_{{{\text{A}}^\prime }} }}{2} + {\text{r}}_{{\text{o}}} }}{{\sqrt 2 \left( {\frac{{{\text{r}}_{{\text{B}}} }}{2} + \frac{{{\text{r}}_{{{\text{B}}^\prime }} }}{2} + {\text{r}}_{{\text{o}}} } \right)}}$$

**Table 1:** Lattice parameters, unit cell volumes, tolerance factors, goodness of fit and crystallite size of the compounds obtained from Rietveld refinement.

Doping content (x)	0.00	0.02	0.04	0.06
Space group	I4/m	I4/m	I4/m	I4/m
Symmetry	Tetragonal	Tetragonal	Tetragonal	Tetragonal
a (Å)	5.5601	5.5631	5.5660	5.5692
b (Å)	5.5601	5.5631	5.5660	5.5692
c (Å)	7.9190	7.9193	7.9230	7.9267
α = β = γ (°)	90	90	90	90
Unit cell volume, V (Å^3^)	244.889	245.090	245.517	245.863
Tolerance factor, τ	0.9820	0.9817	0.9813	0.9810
χ2	1.931	2.737	2.459	3.396
R_p_ (%)	7.67	9.83	8.96	9.90
R_wp_ (%)	9.83	12.80	11.68	11.55
Crystallite size, D (nm)	32.1230	27.254	28.224	28.974

**Table 2:** Bond lengths and bond angles of Sr_2–x_Ca_x_NiWO_6_ perovskite series.

Doping content (x)	0.00	0.02	0.04	0.06
Bond length (Å)				
Ni–O_1_	2.044 (3)	2.046 (8)	2.047 (7)	2.049 (2)
Ni–O_2_	2.019 (3)	2.022 (9)	2.023 (9)	2.024 (6)
< Ni–O >	2.032 (3)	2.034 (8)	2.035 (8)	2.037 (4)
W–O_1_	1.920 (2)	1.922 (7)	1.923 (6)	1.925 (6)
W–O_2_	1.940 (3)	1.943 (7)	1.944 (8)	1.946 (7)
< W–O >	1.930 (2)	1.933 (7)	1.934 (7)	1.936 (6)
Bond angle (°)				
Ni–O_1_–W	155.560 (1)	156.527 (1)	156.676 (1)	157.677 (1)
Ni–O_2_–W	160.657 (1)	161.752 (1)	161.838 (1)	162.897 (1)
** < **Ni–O–W >	158.109 (1)	159.140 (1)	159.257 (1)	160.287 (1)

where r_A_, r_A′_, r_B_, r_B′_ and r_o_ are the ionic radii of A (Sr^2+^), A′(Ca^2+^ ), B (Ni^2+^), B′ (W^6+^) site cations and oxygen ions of double perovskite respectively. To calculate tolerance factors of the compounds, the ionic radii used for these calculations are as follows; Sr^2+^ (1.44 Å), Ca^2+^ (1.34 Å), Ni^2+^ (0.69 Å), W^6+^ (0.60 Å) and O^2-^ (1.40 Å). According to Table 1, the computed tolerance factors for the compounds were 0.9820, 0.9817, 0.9813 and 0.9810. It can be noted that as the Ca^2+^ doping in the compounds increased, the B and B′ octahedra became somewhat distorted causing the compounds’ tolerance factor and stability decreased. In addition, as presented in Table 2, it was observed that the compounds’ bond angles and bond lengths increased slightly as the amount of Ca^2+^ dopants increased, which was associated with the structure’s distortion. The crystallite size (D) of all samples were calculated using the Debye–Scherrer’s equation as follow;

2 $${\text{D}} = \frac{{{\text{K}}\uplambda }}{{\upbeta \cos\uptheta }}$$

where K is a Scherrer constant (0.94), λ is the wavelength of X-ray used (0.15406 nm), β is the full-width half maximum in radian and θ is the angle of most intense peak at (112) in radian. From the calculation, the value of the crystallite sizes of the samples obtained in range of 28.224–32.130 nm.

**Figure 3:**
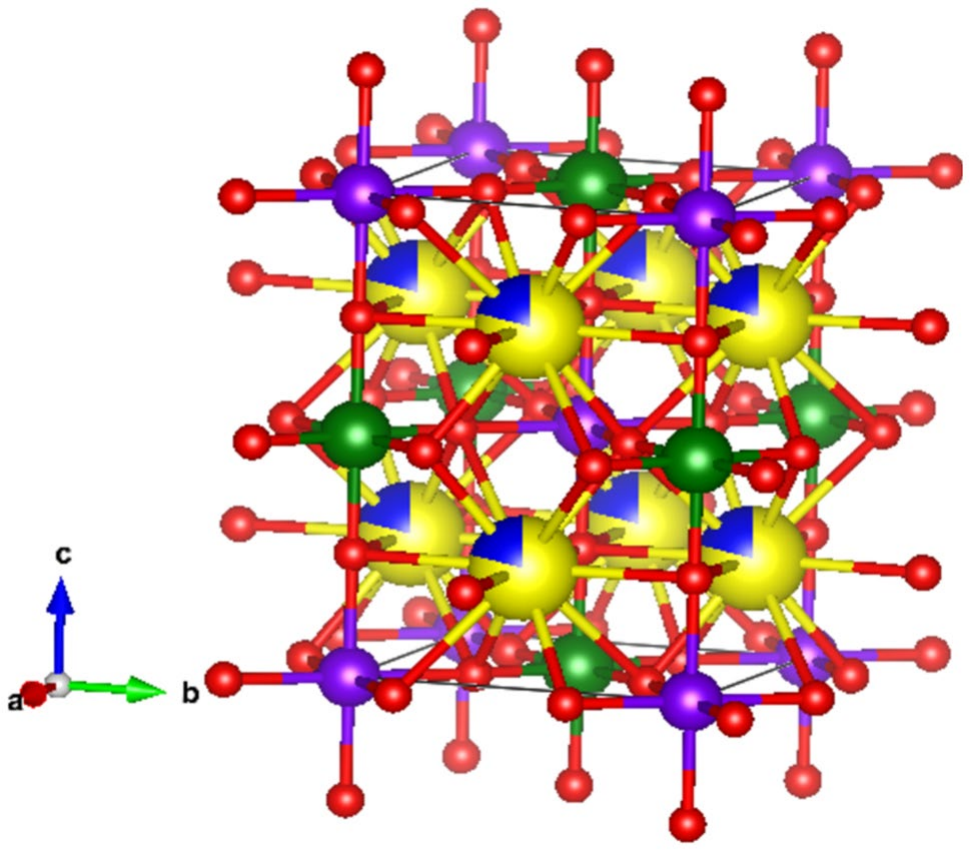
Crystallographic structure of Sr_2–x_Ca_x_NiWO_6_ (x = 0.02). Large yellow spheres, small blue spheres, small purple spheres, small green spheres and red spheres indicate Sr^2+^, Ca^2+^, Ni^2+^, W^6+^ and O^2-^ ions respectively.

Figure 3 shows the crystallographic structure of Sr_2–x_Ca_x_NiWO_6_ (x = 0.02) compound which was constructed using VESTA software. The Ca^2+^ dopants occupied the Sr^2+^ site that was surrounded by 12 oxygen anions meanwhile Ni^2+^ and W^6+^ occupied the B site surrounded by 6 oxygen anions.

### SEM analysis

**Figure 4:**
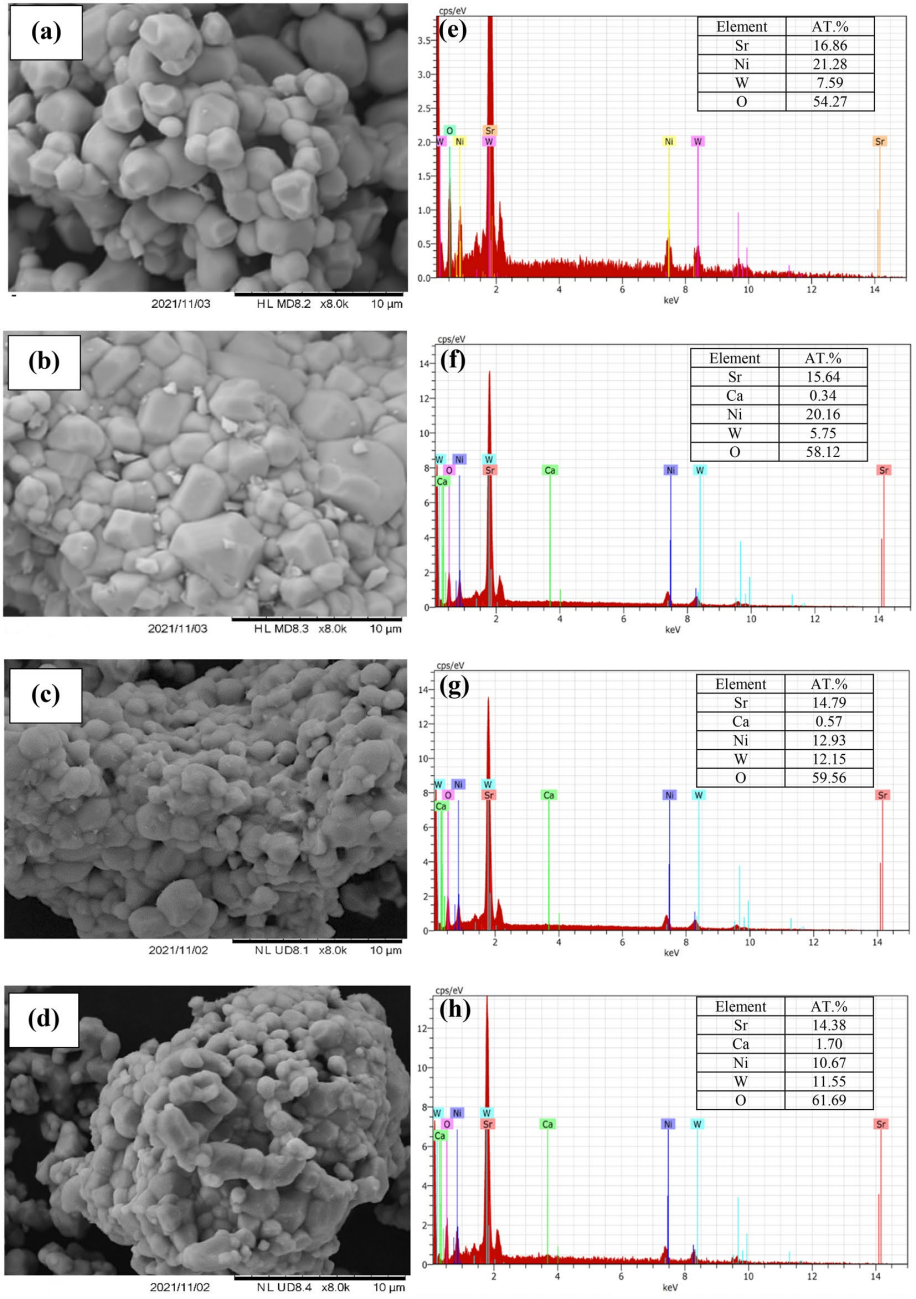
SEM images of Sr_2–x_Ca_x_NiWO_6_ (**a**) x = 0.00, (**b**) x = 0.02, (**c**) x = 0.04, (**d**) x = 0.06 series under 8.0 K magnification and EDX analysis of Sr_2–x_Ca_x_NiWO_6_ (**e**) x = 0.00, (**f**) x = 0.02, (**g**) x = 0.04 and (**h**) x = 0.06.

**Figure 5:**
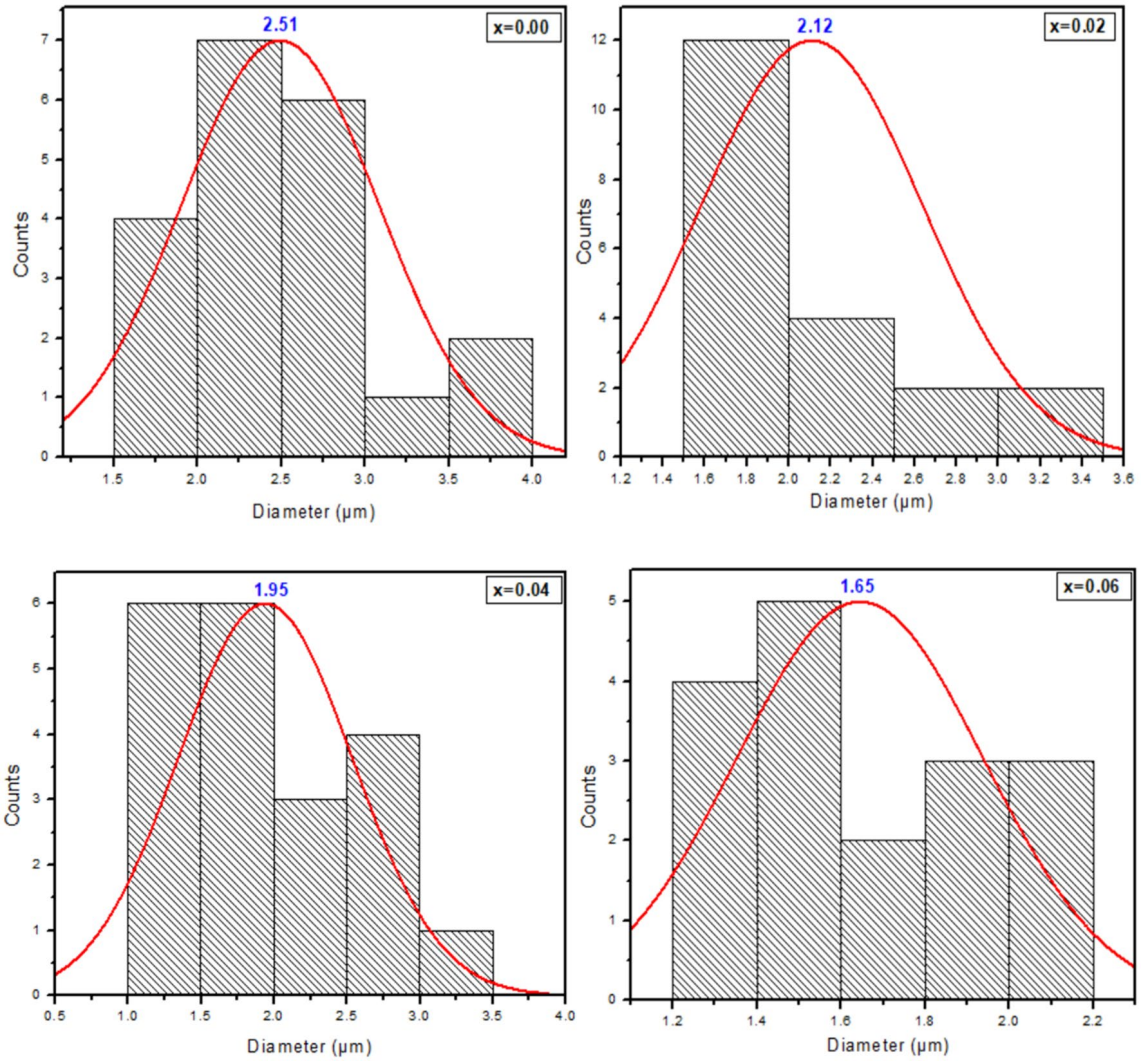
Histograms showing the average grain size distribution of Sr_2–x_Ca_x_NiWO_6_ (x = 0.00, 0.02, 0.04, 0.06) series.

The microstructure of Sr_2–x_Ca_x_NiWO_6_ (x = 0.00, 0.02, 0.04, 0.06) series under 8.0 K magnification are illustrated in Fig. 4. The morphologies of the samples revealed that all compounds existed in irregularly shaped particles with various size of grains. Bijelic et al.^15^, have stated in their report that Sr_2_NiWO_6_ compound formed in irregularly round-shaped particles. Moreover, the particles were also grouped together as a result of higher temperature used during the preparation of the samples. Apart from that, the average grain size of the compounds were determined using Image J software and plotted as histograms depicted in Fig. 5. It can be seen that the average grain size of the samples shrank from 2.51 to 1.65 μm upon Ca^2+^ doping up to x = 0.06. An analysis of energy dispersive X-ray spectroscopy (EDX) was carried out for all samples. All EDX graphs confirmed the presence of all elements Sr^2+^, Ca^2+^, Ni^2+^, W^6+^ and O^2-^ ions in the compounds. It can be clearly seen that the percentage of Sr in parent compound decreased from 16.86% to 14.38% in x = 0.06 which indicated that Ca^2+^ ions has been incorporated at the Sr^2+^ site of the compounds.

### UV–vis analysis

**Figure 6:**
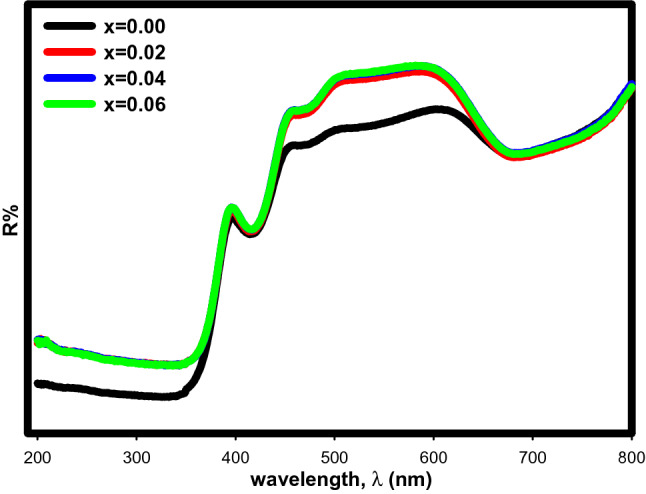
The diffuse reflectance spectra of Sr_2–x_Ca_x_NiWO_6_ (x = 0.00, 0.02, 0.04, 0.06) series.

Figure 6 shows the diffuse reflectance spectra of Sr_2–x_Ca_x_NiWO_6_ (x = 0.00, 0.02, 0.04, 0.06) that were measured at room temperature in the range of 200–800 nm. The strong absorption bands were seen at wavelengths 300–400 nm and 400–500 nm. These bands were connected to the charge transfer between O^2-^ and tungsten, W^6+^ ions from the highest filled molecular orbital 2p oxygen to the lowest vacant molecular orbital 5d tungsten. As Ca^2+^ increased, the compound’s increasing reflectance value revealed a decrease in its absorbance characteristics.

**Figure 7:**
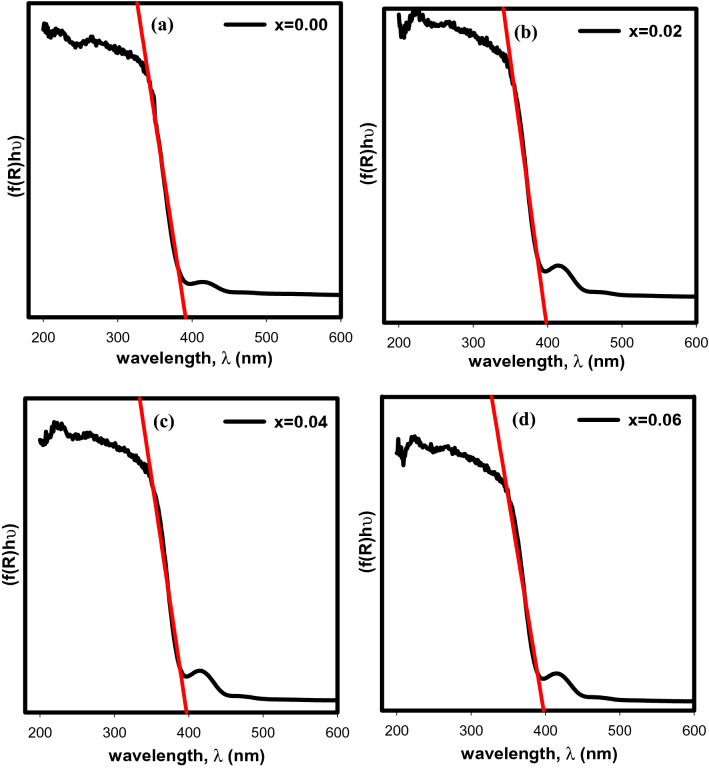
Absorbance vs wavelength of Sr_2–x_Ca_x_NiWO_6_ (**a**) x = 0.00, (**b**) x = 0.02, (**c**) x = 0.04 and (**d**) x = 0.06.

Figure 7 depicts the relationship between the absorbance (f(R)hυ) and wavelength that are plotted using the Kubelka–Munk (KM) function as shown in Eq. (3);

3 $${\text{F }}\left( {{\text{R}}_{\infty } } \right) = { }\frac{\upalpha }{{\text{s}}} = { }\frac{{\left( {1 - {\text{R}}} \right)^{2} }}{{2{\text{R}}}}$$

where F (${\text{R}}_{\infty }$) is the KM function, α is the absorption coefficient, s is the scattering coefficient and R is the reflectance coefficient. The value of the absorption edge obtained from extrapolating the graph to the wavelength axis were 389 nm, 400 nm, 398 nm and 397 nm for x = 0.00, x = 0.02, x = 0.04 and x = 0.06 respectively.

**Figure 8:**
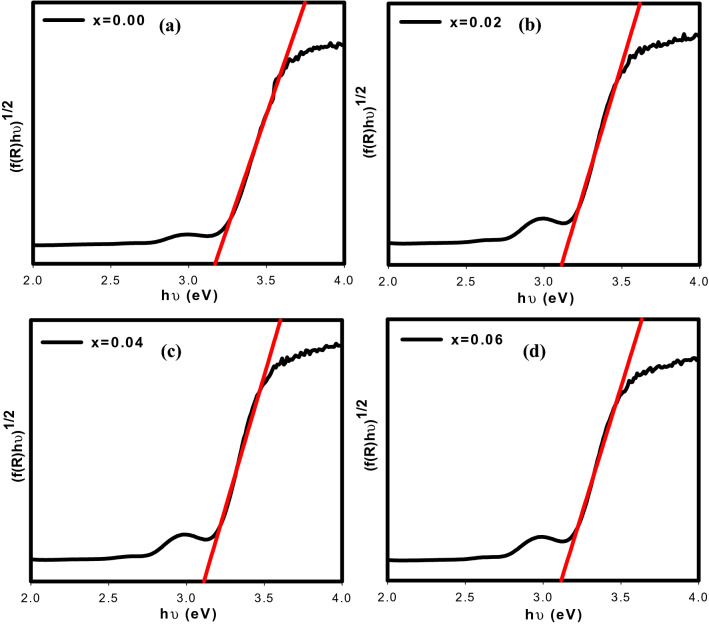
Tauc plot of Sr_2–x_Ca_x_NiWO_6_ (**a**) x = 0.00, (**b**) x = 0.02, (**c**) x = 0.04 and (**d**) x = 0.06.

The band gap energy, E_g_ of the series can be computed from the absorption edge obtained according to the relation of E_g_ = 1240/λ, where λ is absorption edge wavelength. In addition, the E_g_ values also can be directly obtained from the Tauc plot as shown in Fig. 8 using Eq. (4);

4 $$\left( {{\text{F}}\left( {{\text{R}}_{\infty } } \right){\text{h}}\upupsilon } \right)^{{\text{n}}} = {\text{A }}\left( {{\text{h}}\upupsilon - {\text{E}}_{{\text{g}}} } \right)$$

where hυ is the incident photon energy, n can be 1/2, 2, 3/2 or 3 for direct allowed transition, indirect allowed transition, direct forbidden transition and indirect forbidden transition respectively, A is proportional constant and Eg is the band gap energy. By fitting all the values of n in the Tauc relation, it was discovered that n = 1/2 was the best match as it is the most well fitted, leading to direct transitions that enabled electrons to be directly excited from the valence band to conduction band. The band gap energy values derived from the Kubelka–Munk and Tauc plots are shown in Table 3. The band gap energy values calculated from the absorption edge were 3.19, 3.10, 3.11 and 3.12 eV for x = 0.00, x = 0.02, x = 0.04 and x = 0.06 respectively. Meanwhile, the band gap energy values obtained from Tauc plots were 3.17, 3.11, 3.12 and 3.13 eV respectively. According to the findings, the band gap energy obtained using the Kubelka–Munk relations were closely consistent with those acquired from the Tauc plots which decreased with an increase of Ca^2+^ dopant concentration up to x = 0.02. As mentioned by Suraja et al.^21^, the band gap energy of double perovskites typically increased as the ionic radius of A-site substituted cations decreased. The octahedral tilting of the double perovskite causes a reduction in the average bond angles which in turn causes a narrowing of the conduction band width. Therefore, the band gap energies increased. However, in this case, the band gap energies dropped upon Ca^2+^ doping up to x = 0.02 which may be connected to the expansion of the bond angles. In addition, a subsequent upward trend was observed in the band gap energy from x = 0.04 to x = 0.06 which can be related to the decrease of the average particle sizes obtained from SEM analysis. Based on the findings of the optical band gap energy measurements, all samples were classified as semiconductor materials.

**Table 3:** Cut off wavelength, band gap energy of Sr_2–x_Ca_x_NiWO_6_ (x = 0.00, 0.02, 0.04, 0.06) series calculated from Kubelka Munk plot and band gap energy obtained from Tauc plot.

Doping content (x)	0.00	0.02	0.04	0.06
Cut-off wavelength (nm)	389	400	398	397
Band gap energy, E_g_ (eV) from cut-off wavelength	3.19	3.10	3.11	3.12
Band gap energy (eV) obtained from tauc plot	3.17	3.11	3.12	3.13

### Dielectric analysis

**Figure 9:**
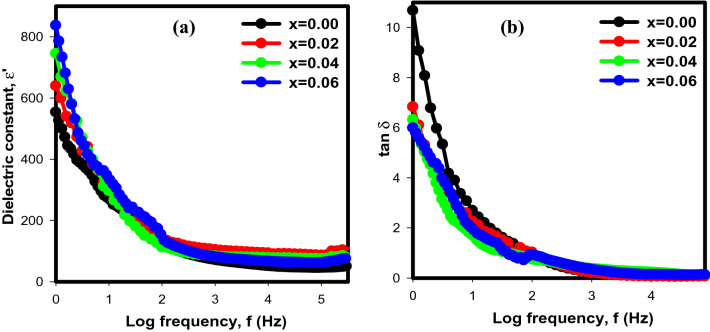
The frequency dependence of (**a**) dielectric constant, ε’ and (**b**) dielectric loss, tan δ for Sr_2–x_Ca_x_NiWO_6_ (x = 0.00, 0.02, 0.04, 0.06) series at room temperature in a frequency range between 1 Hz and 1 MHz.

The dielectric analysis is a very useful technique to study the grain boundary and transport properties, compound structure and charge-storing capacity of materials. Figure 9 shows the frequency dependence of dielectric constant, ε’ and dielectric loss, tan δ for Sr_2–x_Ca_x_NiWO_6_ (x = 0.00, 0.02, 0.04, 0.06) series across the frequency range of 1 Hz to 1 MHz at room temperature. To plot these graph, the ε’ and tan δ values can be determined from Eqs. (5), (6) and (7) respectively;

5 $$\upvarepsilon ^\prime = { }\frac{{ - {\text{Z}}^{\prime{\prime}} }}{{\upomega {\text{C}}_{{\text{o}}} \left( {{\text{Z}}^{\prime 2} + {\text{ Z}}^{\prime \prime 2} } \right)}}$$

6 $$\upvarepsilon ^{\prime \prime } = { }\frac{{{\text{Z}}^\prime }}{{\upomega {\text{C}}_{{\text{o}}} \left( {{\text{Z}}^{\prime 2} + {\text{ Z}}^{\prime \prime 2} } \right)}}$$

7 $$\tan \delta = { }\frac{{\upvarepsilon ^\prime }}{{\upvarepsilon ^{\prime{\prime}} }}$$

where Z′ and Z″ are referred to the real and imaginary part of impedance data obtained from Z View software, ω is angular frequency, C_o_ = (ε_o_A)/d where A = area of the samples and d = thickness of the samples. The graph revealed a consistent patterns for all compositions, with the dielectric constant being larger in the low frequency region (1 Hz). However, as frequency increased up to 1 MHz, the dielectric constant instantaneously decreased until it reached constant levels. The different polarization mechanisms such as ionic, dipolar, electronic and space charge can have a considerable impact on the dielectric constant^22^. At low frequency, the electrical diploes in the compounds line up with the applied electric fields which provides total polarization of the samples. At this region, nearly all polarizations became prominent. However, as the frequency increased to 1 MHz, the contributions of polarizations gradually diminished except for electronic, leading to a low dielectric constant. Hoping electrons are no longer able to follow the rapidly diverging electric field, which alters their motion and lessens the build-up of charges at dielectric material grain boundaries. Thus, the values of dielectric constant decreased. In this study, the decrease in crystallite size from 32.1230 to 28.974 nm may be responsible for the increase in ε’ from ~ 550 in undoped sample, x = 0.00 to ~ 845 in doped sample. As crystallite size decreased the grain boundaries increase which preventing the hopping process between the different states and grains^23^. As a result, the Ca^2+^ ions accumulate at the grain boundaries, raising the resistance and the dielectric constant values. The dielectric loss, tan δ which denotes the energy lost during the alternation of the applied electric field is another crucial aspect of dielectric analysis. The variation of the dielectric loss with responds to the frequency in Fig. 9b can be explained using the Maxwell–Wagner two layers theory which asserts that the samples are composed of two layers, namely grains and grains boundaries^24^. Based on this theory, the grain boundaries are more active at low frequency region. As a result, the build-up of charge carriers keeps growing. Due to their high resistance, more energy is required to transport the charge carriers across the grain boundaries and resulting in high dielectric loss. However, in the high frequency range, the grains are quite active, allowing charge carriers to flow at the grain more easily without dissipating much energy. Thus, the dielectric loss is low.

### Impedance spectroscopy analysis

**Figure 10:**
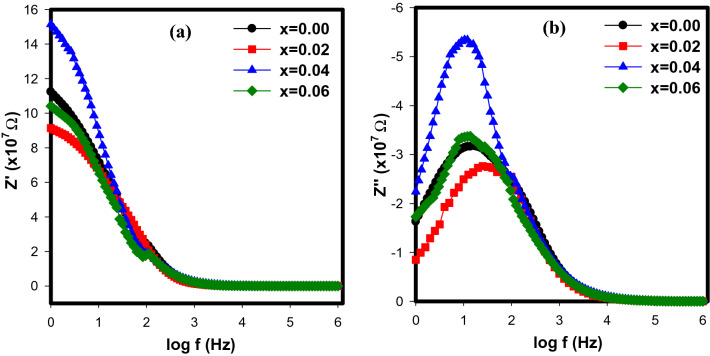
(**a**) Z’ and (**b**) Z’’ with frequency of Sr_2–x_Ca_x_NiWO_6_ (x = 0.00, 0.02, 0.04, 0.06) series at room temperature 300 K.

The complex impedance (Z) has been examined to evaluate the resistive or capacitive contribution to the material’s conductivity after applying an alternating current electric field. Figure 10a,b show the results of the complex impedance measurement of the double perovskite Sr_2–x_Ca_x_NiWO_6_ (x = 0.00, 0.02, 0.04, 0.06) at a frequency range of 1 Hz to 1 MHz. All Z′ values were found to be stable at lower frequencies and to decrease as frequency rose. The decrease in Z’ values shows that the compounds’ AC conductivity has risen^25^. According to the graph, the fact that all of the Z′ curves converged at about 90 kHz shows that the space charges were liberated which led to an increase in the compounds’ AC conductivity. The graph of imaginary component of impedance (Z″) vs frequency is shown in Fig. 9b. A single peak was present in this plot, signifying the survival of one dielectric relaxation process in the double perovskite compounds. This process appears is due to the interface effect^25^.

**Figure 11:**
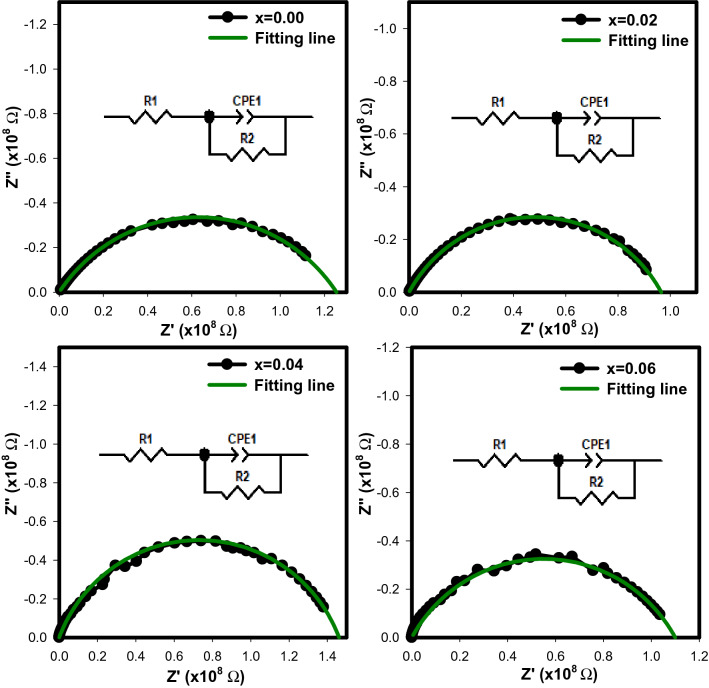
Nyquist plot of Sr_2–x_Ca_x_NiWO_6_ (x = 0.00, 0.02, 0.04, 0.06) series. The internal figures indicate the equivalent circuit obtained for each compounds.

The Nyquist plot describes the variation of Z′ with Z″ in the frequency range between 1 Hz to 1 MHz for Sr_2–x_Ca_x_NiWO_6_ (x = 0.00, 0.02, 0.04, 0.06) series are shown in Fig. 11. The Nyquist plot, in general, has a few semi-circular arcs with the first circle at higher frequency depicting the grain/bulk effect. The influence of the grain boundary is shown by the second circle, which operates at intermediate frequency, meanwhile the third circle at low frequency area represents the electrode effect. In this case, the plots consist only a single semi-circular arc that appeared at higher frequencies indicating the electrical properties of these compounds were attributed to the grain/bulk effect. The scattered symbols which represented the experimental data were fitted using Z View software to identify an equivalent circuit model for these compounds. The equivalent circuits of resistance and constant phase element (CPE) yielded parameters such as grain resistance, R_g_ and grain capacitance, C_g_ and listed in Table 4. It was observed that R_g_ values decreased from 1.2458 × 10^8^ Ω (x = 0.00) to 1.0944 × 10^8^ Ω (x = 0.06) and C_g_ values increased from 0.4298 × 10^–10^ F (x = 0.00) to 0.8115 × 10^–10^ F (x = 0.06). The compounds thus exhibited semiconducting behaviour and might be used in energy storage devices^24^. Previous studies stated that when Debye-type conduction takes place, the semi-circular centre is on the real Z′ axis. In contrast, when non-Debye type conduction occur, the centre is positioned below the real Z′ axis, creating in a depression angle. The Nyquist plot for all compounds confirmed a non-Debye type due to the deviation of the impedance plane from the real axis by a certain angle called depression angle^25,26^.

**Table 4:** Values of grain resistance (R_g_) and grain capacitance (C_g_) obtained from the fitting of Nyquist plot with R-CPE equivalent circuit model.

Doping content (x)	R_g_ (10^8^ Ω)	C_g_ (10^–10^ F)
0.00	1.2458	0.4298
0.02	0.9629	0.4836
0.04	1.4655	1.1230
0.06	1.0944	0.8115

## Conclusions

In conclusion, the Sr_2–x_Ca_x_NiWO_6_ (x = 0.00, 0.02, 0.04, 0.06) double perovskites were synthesized using a solid state reaction method. All compounds were crystallized in a tetragonal structure with a space group I4/m. The SEM analysis showed that all compounds existed in irregularly shaped particles and agglomerated in groups. In addition, the grain sizes of the compounds decreased as Ca content increased. The UV–vis analysis revealed that the value of the band gap energy decreased. All samples were classified as wide bandgap semiconductor materials. The dielectric analysis revealed that the dielectric constant and loss were higher at low frequency region and decreased at high frequency region. Sr_1.94_Ca_0.06_NiWO_6_ exhibited the highest dielectric constant compared to the parent compound, Sr_2_NiWO_6_ which depicts that the doping itself may change the dielectric properties of double perovskite compounds.

## Data Availability

The datasets generated during and/or analyzed during the current study are available from the corresponding author, Z.M. on reasonable request.
